# Performance of Plasma Biomarkers Combined with Structural MRI to Identify Candidate Participants for Alzheimer’s Disease-Modifying Therapy

**DOI:** 10.14283/jpad.2024.110

**Published:** 2024-06-21

**Authors:** M. Manjavong, J. M. Kang, A. Diaz, M. T. Ashford, J. Eichenbaum, A. Aaronson, M. J. Miller, S. Mackin, R. Tank, M. Weiner, Rachel L. Nosheny, Alzheimer’s Disease Neuroimaging Initiative

**Affiliations:** 1 Division of Geriatric Medicine, Department of Internal Medicine, Faculty of Medicine, Khon Kaen University, Khon Kaen, Thailand; 2 Department of Psychiatry, Gil Medical Center, Gachon University College of Medicine, Incheon, Republic of Korea; 3 Department of Radiology and Biomedical Imaging, University of California San Francisco, San Francisco, CA USA; 4 Northern California Institute for Research and Education (NCIRE), San Francisco, CA USA; 5 Department of Veterans Affairs Medical Center, VA Advanced Imaging Research Center, San Francisco, California USA; 6 Department of Psychiatry, San Francisco VA Medical Center, University of California, San Francisco, 4150 Clement Street (114M), San Francisco, CA 94121 USA; 7 Dementia Research Centre, UCL Institute of Neurology, University College London, London, WC1E 6BT UK; 8 Department of Neurology, University of California San Francisco, San Francisco, CA USA; 9 Department of Medicine, University of California San Francisco, San Francisco, CA USA

**Keywords:** Plasma biomarkers, Alzheimer’s disease, plasma Aβ42/Aβ40, plasma pTau181, plasma NFL

## Abstract

**Background:**

Recently, two monoclonal antibodies that lower amyloid plaques have shown promising results for the treatment of Mild Cognitive Impairment (MCI) and mild dementia due to Alzheimer’s disease (AD). These treatments require the identification of cognitively impaired older adults with biomarker evidence of AD pathology using CSF biomarkers or amyloid-PET. Previous studies showed plasma biomarkers (plasma Aβ42/Aβ40 and p-tau181) and hippocampal volume from structural MRI correlated with brain amyloid pathology. We hypothesized plasma biomarkers with hippocampal volume would identify patients who are suitable candidates for disease-modifying therapy.

**Objectives:**

To evaluate the performance of plasma AD biomarkers and hippocampal atrophy to detect MCI or AD with amyloid pathology confirmed by amyloid-PET or CSF biomarkers in ADNI.

**Design:**

A cross-sectional and longitudinal study.

**Setting and Participants:**

Data were from the Alzheimer’s Disease Neuroimaging Initiative. Participants were aged 55–90 years old with plasma biomarker and structural MRI brain data.

**Measurements:**

The optimum cut-off point for plasma Aβ42/Aβ40, p-tau181, and NFL and the performance of combined biomarkers and hippocampal atrophy for detecting cognitive impairment with brain amyloid pathology were evaluated. The association between baseline plasma biomarkers and clinical progression, defined by CDR-Sum of Boxes (CDR-SB) and diagnostic conversion over two years, was evaluated using a Weibull time-to-event analysis.

**Results:**

A total of 428 participants were included; 167 had normal cognition, 245 had MCI, and 16 had mild AD. Among MCI and AD, 140 participants had elevated amyloid levels by PET or CSF. Plasma Aβ42/Aβ40 provided the best accuracy (sensitivity 79%, specificity 66%, AUC 0.73, 95% CI 0.68–0.77) to detect drug candidate participants at baseline. Combined plasma Aβ42/40, p-tau181, and hippocampal atrophy increased the specificity for diagnosis (96%), but had lower sensitivity (34%), and AUC (0.65). Hippocampal atrophy combined with the abnormal plasma p-tau181 or hippocampal atrophy alone showed high sensitivity to detect clinical progression (by CDR-SB worsening) of the drug-candidate participants within the next 2 years (sensitivity 93% and 89%, respectively).

**Conclusion:**

Plasma biomarkers and structural MRI can help identify patients who are currently eligible for anti-amyloid treatment and are likely to progress clinically, in cases where amyloid-PET or CSF biomarkers are not available.

**Electronic Supplementary Material:**

Supplementary material is available in the online version of this article at 10.14283/jpad.2024.110.

## Introduction

**A**lzheimer’s disease (AD) is a neurodegenerative disease pathologically defined by amyloid plaques, tau tangles, and neurodegeneration (1). AD causes progressive cognitive impairment and is the major cause of dementia in the elderly (1, 2). AD patients usually present with a progressive decline in their cognition, which is severe enough to cause functional impairment (3).

Novel, disease modifying medications are initiating a new era for AD treatment. U.S. Food and Drug Administration approved Aducanumab in 2021 via the Accelerated Approval pathway and Lecanemab in 2023 via Traditional FDA Approval for treating AD. Recently, Donanemab provided positive results in a randomized clinical trial to slow the clinical progression of AD patients (4–6). The medications mentioned are designed to target the underlying causes of AD by removing Amyloid-beta (Aβ) plaques. Lecanemab has been approved for the early stages of AD (mild cognitive impairment (MCI) and mild AD dementia), with evidence for elevated brain amyloid (4, 7). Identifying older adults who are suitable candidates for AD therapeutics is crucial in ensuring patients receive prompt treatment.

Amyloid PET or lumbar puncture for CSF amyloid are needed to confirm whether patients should receive AD therapies. However, these procedures are invasive, time-consuming, and costly and have some limitations, such as counterindication in patients with coagulopathy. Therefore, we investigated whether more efficient methods, including structural MRI and plasma biomarkers, could be used to identify patients who are likely to benefit from AD therapeutics.

According to previous studies, numerous plasma biomarkers are associated with the clinical and pathological sequelae of AD. Decreasing plasma Aβ of 42 amino acids/ Aβ of 40 amino acids (Aβ1–42/Aβ1–40) ratio (8–10) and increasing plasma total tau and p-tau (phosphorylated tau) levels correlate with abnormal Aβ and tau protein deposition in PET scans of MCI and AD patients (11) and differentiate diagnostic groups, including cognitively unimpaired (CU) vs early AD (12). Moreover, plasma Aβ42/Aβ40 and plasma phosphorylated-tau181 (p-tau181) significantly correlated with CSF Aβ42 and CSF p-tau181, respectively (13), and p-tau181 was associated with cognitive decline (14) and can distinguish cognitively impaired participants from cognitively normal participants (15).

For the relationship between structural MRI brain and AD, evidence shows rapid loss of hippocampal volume in the early stage of AD. Subsequently, medial temporal and hippocampal atrophy occur with disease progression in AD (16). Furthermore, a previous study has shown an association between lower levels of plasma Aβ42 and a decrease in the volume of the hippocampus (17). Apart from plasma Aβ and p-tau, neurofilament light protein (NFL), which indicates neurodegeneration, has shown an association with hippocampal atrophy in AD patients (18). A prior study found that combining hippocampal volume and serum NFL with plasma Aβ and p-tau could enhance the predictive value for progression to MCI and dementia (19).

Most previous studies compared the associations of each plasma biomarker separately with clinical or imaging outcomes, and some of them defined MCI or AD by using clinical characteristics without amyloid pathology confirmation (14, 20). The primary objective of this study was to assess the performance of combined plasma biomarkers, including plasma Aβ42/40 ratios, plasma p-tau181, and plasma NFL to identify older adults who are suitable candidates to receive AD therapeutics: early-stage AD patients (those with MCI or mild AD) with positive amyloid pathology. The secondary objective was to evaluate the ability of plasma biomarkers at baseline to predict which drug-candidate participants are at risk for progressive cognitive decline within 2 years. This is important because it can both identify older adults most likely to benefit from therapeutic intervention, and facilitate future AD clinical trials by informing participant selection. Since structural MRI is often available as part of a routine clinical workup, we further investigated whether structural MRI improved the ability to identify AD drug candidates.

## Methods

### Subjects and study setting

This study included both cross-sectional and longitudinal analyses. Data used to prepare this article were obtained from the Alzheimer’s Disease Neuroimaging Initiative (ADNI) database (https://adni.loni.usc.edu/). The ADNI was launched in 2004 as a public-private partnership led by Principal Investigator Michael W. Weiner, MD. The primary goal of ADNI has been to test whether serial magnetic resonance imaging (MRI), positron emission tomography (PET), other biological markers, and clinical and neuropsychological assessment can be combined to measure the progression of mild MCI and early AD. Data in this study was derived from phases ADNI 1, ADNI GO, ADNI 2, and ADNI 3. Participants were aged between 55–90 years. You can download the complete list of eligibility criteria, including both inclusion and exclusion criteria, from the following URL https://adni.loni.usc.edu/methods/documents/.

### Procedure

At baseline enrollment, all participants were evaluated by the clinicians of each study site. Their baseline characteristics including age, gender, race, and comorbidities were collected. They completed assessments including ADAS-Cog, MoCA, Neuropsychological battery, Everyday Cognition Scale, and Activities of Daily Living. They were diagnosed as cognitively unimpaired, mild MCI, or mild AD by clinicians’ judgment. All participants included in this analysis underwent testing for plasma biomarkers, genetic analysis, structural MRI, Amyloid PET imaging, and CSF profiles. All participants would be monitored on their blood biomarkers and neuropsychological testing regularly for at least two years.

### Diagnostic Groups

#### Cognitively unimpaired

Participants were without any memory complaints common to their age range. The neuropsychological and functional tests revealed normal results. Cognitively normal, based on an absence of significant impairment in cognitive functions or activities of daily living.

#### MCI

Participants who either self-reported memory complaints or had complaints reported by their study partners, had abnormal memory function based on scoring below the education-adjusted cutoff on the Logical Memory II subscale from the Wechsler Memory Scale-Revised. However, their cognition and functional performance were sufficiently preserved that they did not meet the criteria for dementia due to Alzheimer’s Disease. The MCI participants were divided into amyloid-positive and amyloid-negative MCI. The amyloid-positive MCI was defined by meeting the criteria for MCI with evidence of amyloid pathology (via amyloid-PET scan or CSF biomarkers), while the term «amyloid-negative MCI» refers to cases of MCI where no signs of amyloid pathology are observed.

#### Alzheimer’s disease (AD)

Participants who either self-reported memory complaints or had complaints reported by their study partners and had abnormal memory function based on scoring below the education-adjusted cutoff. Their clinical met the criteria for probable AD by NINCDS/ADRDA. The Clinical Dementia Rating global score was 0.5 or 1. The amyloid-positive AD was defined by meeting the criteria for AD with evidence of amyloid pathology (via amyloid-PET scan or CSF biomarkers), while the term «amyloid-negative AD» refers to cases of AD where no signs of amyloid pathology are observed.

#### Drug Candidate Determination

Participants who are suitable candidates for receiving anti-amyloid monoclonal antibody AD therapeutics were defined as MCI or AD participants with positive evidence of amyloid pathology confirmed by amyloid-PET scan or CSF biomarkers.

#### Non-drug Candidate Determination

Non-drug candidate participants included cognitively unimpaired participants and MCI or AD participants who did not have amyloid pathology evidence from amyloid-PET scan or CSF biomarkers.

### Clinical progression

In drug candidate participants, we evaluated the association between baseline plasma biomarkers and clinical progression within the next two years. For the time-to-event analysis, we defined clinical progression in two ways.

#### Defined by diagnostic conversion

Participants were considered to have progressed if they had an MCI diagnosis at baseline and at least one subsequent visit met the criteria for AD dementia.

#### Defined by change in CDR-SB score (21)

Participants were considered to have progressed if (1) Participants fulfilled MCI criteria at baseline, and their Clinical Dementia Rating Scale-Sum of Boxes (CDR-SB) score worsened by at least 1 point from their initial assessment in two consecutive visits, or (2) Participants fulfilled AD criteria at baseline and experienced a decline of 2 or more points in their CDR-SB score at two consecutive visits compared to their initial assessment. According to a prior study, a 1 to 2-point difference in CDR-SB indicates a clinically significant change in MCI and AD, respectively (21).

### Evidence of amyloid pathology

Evidence of amyloid pathology in this study was defined using an amyloid-PET scan or CSF biofluids. First, we analyzed an amyloid-PET scan’s standardized uptake value ratio (SUVR) converted to the Centiloid scale (CL) to harmonize data using the formula CL=180.20 × ADNI FBP SUVR – 179.70 (22). The presence of amyloid pathology was defined as CL greater than or equal to 18.5 (22). In case of no PET scan results, CSF biomarkers were used to identify evidence of amyloid pathology. Evidence of amyloid pathology was defined by CSF Aβ42 < 980 pg/mL and ptau181/Aβ42 ratio ≥ 0.025 (23–25).

### Plasma Biomarkers

Plasma Aβ40 and Aβ42 were measured by immunoprecipitation in the Bateman Laboratory and subsequently handled with Lys-N protease and liquid chromatography-tandem mass spectrometry (LC-MS/MS) (26). The plasma p-tau181 and NFL were measured using the single-molecule array (SIMOA) technique from the Clinical Neurochemistry Lab at the University of Gothenburg, Sweden (27).

### Structural MRI

Structural MRI was performed using a Trio 3.0 T scanner or Vision 1.5 T scanner (GE, Siemens, and Philips) to evaluate total hippocampal volume, data can be downloaded from ADNI database website (https://adni.loni.usc.edu/).

### Statistical analysis

Descriptive statistics describing the demographic and clinical characteristics of the cohort are presented in Table 1. Participants were classified as potential candidates for anti-amyloid therapy intervention on the basis of having MCI or AD with positive evidence of Amyloid pathology confirmed by amyloid-PET scan or CSF biomarkers. Two separate cutpoints were estimated for each of hippocampal volume, plasma Aβ42/40 ratio, plasma Ptau181, and plasma NFL): one cutpoint based on the ability to distinguish drug candidates from the remaining cohort, and one cutpoint for distinguishing high-risk drug candidates – those who experienced clinical diagnostic progression within two years of enrollment in the study – from those who did not undergo clinical progression within that two-year window. Optimal cutpoints were estimated using robust nonparametric smoothing methods (28), and in-sample performance of the cutpoints was evaluated using univariate logistic regressions.

**Table 1:** Baseline characteristic

**Characteristics**	**Non-drug candidate (n = 288)**	**Drug candidate (n = 140)**	**P-value**
Age; mean (SD)	71 (7)	73 (7)	0.043
Male; n (%)	143 (50%)	79 (56%)	0.2
Education years; median (IQR)	16 (15, 18)	16 (14, 18)	0.2
Race; n (%)			0.8
- American Indian/ Alaskan	2 (0.7%)	0	
- Asian	6 (2.1%)	2 (1.4%)	
- Black	9 (3.1%)	3 (2.1%)	
- White	265 (92%)	130 (93%)	
- More than one	5 (1.7%)	5 (3.6%)	
- Unknown	1 (0.3%)	0	
Diagnosis			<0.001
- Cognitively unimpaired	167 (58%)	0	
- MCI	120 (41.6%)	125 (89%)	
- Dementia	1 (0.3%)	15 (11%)	
Number of APOE-e4 alleles; n(%)			< 0.001
- 0	207 (72%)	49 (35%)	
- 1	74 (26%)	66 (47%)	
- 2	7 (2.4%)	25 (18%)	
Plasma Aβ42/Aβ40; median (IQR)	0.124 (0.116, 0.132)	0.113 (0.107, 0.119)	< 0.001
Plasma p-tau181; median (IQR)	13 (9, 18)	19 (14, 26)	< 0.001
Plasma NFL; median (IQR)	30 (23, 39)	38 (28, 47)	< 0.001
Hippocampal volume; median (IQR)	7521 (6921, 8062)	6792 (6153, 7614)	< 0.001
Baseline MMSE; median (IQR)	29 (28, 30)	28 (26, 29)	< 0.001
Baseline CDRSB; median (IQR)	0 (0,1)	1.5 (1, 2.5)	< 0.001

MCI, mild cognitive impairment; CDR-SB, Clinical Dementia Rating Scale sum of box score; MMSE, Mini-Mental State Examination; IQR, interquartile range; P-values represent differences between diagnostic groups based on the Kruskal-Wallis rank sum test for continuous variables or the Chi-square test for categorical variables. P-values < 0.05 indicate significant differences.

Time-to-progression was calculated for participants who underwent clinical progression defined by CDRSB progression and diagnostic change, and a multivariable Weibull accelerated failure time (AFT) model was fit to examine the association between the biomarkers of interest and time-to-progression.

## Results

### Participants

There are 428 participants with plasma biomarker data: 167 were unimpaired (CU), 245 were diagnosed with MCI, and 16 had mild AD. The mean age of participants was 71.79 years old. The mean age of CU, MCI, and AD participants were 73.16, 70.53, and 76.67 years old, respectively. In the CU, MCI, and AD groups, 58 (34.73%), 125 (51.02%), and 15 (93.75%) individuals, respectively, showed evidence of amyloid pathology. Baseline characteristics comparing drug candidate and non-drug candidate participants are shown in Table 1.

### Ability to distinguish groups

The performance of each plasma biomarker, combined plasma biomarkers, and hippocampal atrophy to differentiate drug candidates from non-drug candidate participants were shown in Table 2. Plasma p-tau181, plasma NFL, and hippocampal volume from structural MRI showed poor performance in detecting drug-candidate participants (AUC 0.62–0.67), while plasma Aβ42/Aβ40 provided moderate performance (AUC 0.73). A model containing all biomarkers exhibited poor predictive performance but a very high level of specificity.

**Table 2:** Performance of biomarker cutpoints to detect drug candidates at baseline

	**Cut of point**	**Sensitivity**	**Specificity**	**AUC (95% CI)**	**PPV**	**NPV**	**Accuracy**
Plasma Aβ42/Aβ40	≤ 0.12	0.79	0.66	0.73 (0.68–0.77)	0.53	0.88	0.7
Plasma p-tau181	≥ 16.5	0.63	0.72	0.67 (0.62–0.72)	0.52	0.8	0.69
Plasma NFL	≥ 37.5	0.53	0.72	0.62 (0.57–0.67)	0.48	0.76	0.66
Hippocampal volume	≤ 6868	0.54	0.77	0.65 (0.6–0.71)	0.53	0.78	0.69
Aβ42/40 ratios + p-tau181+ PNFL (n =428)	-	0.31	0.93	0.62 (0.58–0.66)	0.67	0.73	0.72
Aβ42/40 ratios + p-tau181 + PNFL+ hippocampal atrophy (n=383)	-	0.26	0.98	0.62 (0.58–0.66)	0.86	0.73	0.75
Aβ42/40 ratios + p-tau181+ hippocampal atrophy (n=383)	-	0.34	0.96	0.65 (0.61–0.69)	0.81	0.75	0.76

AUC: Area under the ROC curve; PPV: Positive Predictive Value; NPV: Negative Predictive Value

### Clinical Progression

Out of the 140 participants in the drug candidate group, 123 were evaluated at least twice using CDR-SB within a 2-year follow-up period. Sixty-four participants (52%) had cognitive worsening by CDR-SB criteria. CDR-SB scores with IQR of the non-progressed and progressed participants at baseline were 1 (0.5, 2) and 1.75 (1, 2.5), respectively. In the progressor group, the median survival time was 388 days (IQR 214, 734 days). The baseline characteristic and baseline plasma biomarkers compared between progressors and non-progressors by CDR-SB worsening criteria were presented in Appendix 1.

### Ability to predict future clinical progression

To detect progressors within the drug candidate participants, hippocampal atrophy from structural MRI showed better performance than plasma biomarkers to detect progressors defined from both clinical diagnosis changes and CDR-SB worsening with AUC 0.71 and 0.62, respectively (Tables 3 and 4). Among plasma biomarkers, plasma p-tau181 provided the best performance to detect disease progression defined by clinical diagnostic change (AUC 0.62) and CDRSB progression (AUC 0.6). Adding plasma p-tau181 to structural MRI slightly increased the performance (AUC 0.62 to 0.66) to predict progression within 2 years, defined by clinical diagnostic change. Moreover, hippocampal atrophy alone (sensitivity 89%) and combining plasma p-tau181 with hippocampal atrophy (sensitivity 93%) had high sensitivity to detect progressors by CDRSB worsening.

**Table 3:** Performance of biomarker cutpoints at baseline to detect disease progression of the drug candidate participants by clinical diagnosis change

	**Cut of point**	**Sensitivity**	**Specificity**	**AUC (95% CI)**	**PPV**	**NPV**	**Accuracy**
Plasma Aβ42/Aβ40	≤ 0.1	0.09	0.91	0.5 (0.44–0.56)	0.27	0.73	0.69
Plasma p-tau181	≥ 27.1	0.36	0.88	0.62 (0.53–0.71)	0.52	0.79	0.74
Plasma NFL	≥ 38	0.58	0.57	0.57 (0.47–0.67)	0.33	0.78	0.57
Hippocampal volume	≤ 5980	0.47	0.95	0.71 (0.62–0.79)	0.79	0.81	0.81
Plasma p-tau181 + hippocampal atrophy Both vs none* (n= 76)	-	0.31	1	0.66 (0.54–0.77)	1	0.85	0.86
Plasma p-tau181 + hippocampal atrophy Both vs other † (n=109)	-	0.15	1	0.58 (0.51–0.64)	1	0.74	0.75

AUC: Area under the ROC curve; PPV: Positive Predictive Value; NPV: Negative Predictive Value; *Both vs none: distinguish between participants who had both high plasma p-tau181 and hippocampal atrophy vs participants who had normal plasma p-tau181 and hippocampal volume; † Both vs other: distinguish between participants who had both high plasma p-tau181 and hippocampal atrophy vs other participants (had normal plasma p-tau181 and hippocampal volume or had only one of each high plasma p-tau181 or hippocampal atrophy)

**Table 4:** Performance of biomarker cutpoints at baseline to detect disease progression of the drug candidate participants by CDRSB progression

	**Cut of point**	**Sensitivity**	**Specificity**	**AUC (95% CI)**	**PPV**	**NPV**	**Accuracy**
Plasma Aβ42/Aβ40	≤ 0.11	0.31	0.49	0.6 (0.52–0.68)	0.39	0.41	0.4
Plasma p-tau181	≥ 16.6	0.71	0.49	0.6 (0.52–0.68)	0.59	0.63	0.6
Plasma NFL	≥ 52.4	0.24	0.91	0.58 (0.52–0.64)	0.74	0.54	0.58
Hippocampal volume	≤ 7629	0.89	0.36	0.62 (0.55–0.7)	0.6	0.75	0.63
p-tau181 + hippocampal atrophy Both vs none * (n= 76)	-	0.93	0.38	0.66 (0.57–0.74)	0.63	0.83	0.67
p-tau181 + hippocampal atrophy Both vs other t (n=109)	-	0.64	0.59	0.62 (0.53–0.7)	0.63	0.6	0.62

AUC: Area under the ROC curve; PPV: Positive Predictive Value; NPV: Negative Predictive Value; *Both vs none: distinguish between participants who had both high plasma p-tau181 and hippocampal atrophy vs participants who had normal plasma p-tau181 and hippocampal volume; † Both vs other: distinguish between participants who had both high plasma p-tau181 and hippocampal atrophy vs other participants (had normal plasma p-tau181 and hippocampal volume or had only one of each high plasma p-tau181 or hippocampal atrophy)

### Time-to-progression

The results of the Weibull analysis are shown in Table 5, with associations shown as both acceleration factors and hazard ratios. Plasma NFL and Aβ42/Aβ40 ratio were associated with greater risk of progression among drug candidates by CDRSB criteria; HR 1.02 (95% CI 1–1.03) and 1.4 (95% CI 1.1–1.79). Baseline hippocampal volume and age were both associated with decreased risk of clinical progression; HR 0.97 (95% CI 0.94–0.99) and 0.93 (95% CI 0.89–0.97).

**Table 5:** Association between plasma biomarkers, hippocampal atrophy, and cognitive impairment progression by CDRSB worsening criteria among drug candidate participants

	**AF**	**95% CI**	**HR**	**95% CI**	**P-value**
Plasma Aβ42/Aβ40 (every 0.01 unit increasing)	0.85	0.75–0.95	1.4	1.1–1.79	0.007
Plasma NFL	0.99	0.98–0.99	1.02	1–1.03	0.04
Hippocampal volume (every 100 mm^3^ of increasing volume)	1.02	1.001–1.03	0.97	0.94–0.99	0.03
Intracerebral volume	0.99	0.99–1	1	0.99–1	0.67
Age	1.04	1.01–1.06	0.93	0.89-0.97	0.002

AF: Acceleration Factor; HR: Hazard ratio

## Discussion

The major findings were: 1) The plasma Aβ42/Aβ40 ratio had the highest AUC (0.73) and highest sensitivity (0.79) among plasma biomarkers to distinguish drug candidate participants from non-candidates at baseline. 2) Aβ42/Aβ40 ratio combined with plasma p-tau181, plasma NFL, and structural imaging together showed a very good specificity in distinguishing groups. 3) Plasma Aβ42/Aβ40 ratio, plasma pTau181, and plasma NFL had poor performances (AUC 0.5–0.62) in predicting clinical progression (both CDRSB progression and clinical diagnostic change). However, combined plasma p-tau181 and baseline hippocampal atrophy had high sensitivity (93%) but low specificity (38%) in predicting cognitive deterioration by CDRSB score progression. 4) Increasing plasma NFL associated with risk of clinical progression in drug-candidate participants. Taken together, these results support the use of plasma biomarkers and structural MRI measures for helping to identifying and prioritizing older adults to receive anti-amyloid therapeutics in many clinical settings, such as geriatric clinics.

Past studies have shown a strong relationship between plasma Aβ42/Aβ40 ratio and brain amyloidosis measured by PET imaging or CSF biomarkers (29, 30). Our results extend these findings by investigating the ability of plasma Aβ42/Aβ40 ratio to distinguish older adults who would or would not be suitable candidates to receive approved AD medications. For other plasma biomarkers, the current study’s results showed that plasma p-tau181 and plasma NFL had fair performance in identifying amyloid-positive participants with cognitive impairments (AUC 0.67 and 0.62, respectively). These can be explained by the fact that NFL is not a specific biomarker of AD, but rather a biomarker of neurodegeneration. For plasma p-tau181, our results are consistent with the Coomans, et al. who showed that plasma pTau181 had high accuracy in identifying Aβ pathology in the preclinical stage of dementia (AUC 0.83; 95% CI 0.7–0.96); however, to differentiate cognitive disease staging, plasma pTau181 (AUC 0.74) had lower performance than Tau PET imaging at temporal region (AUC 0.92) (31).

The result of plasma Aβ42/Aβ40 performance from our study, which showed 79% sensitivity and 66% specificity to detect Aβ-positive cognitive impairment at baseline, supported that plasma Aβ42/Aβ40 testing in cognitively impaired patients may be a convenient and non-invasive screening test to select candidates to receive AD drugs. Moreover, combining plasma p-tau181 and evidence of hippocampal atrophy from structural MRI with or without evidence of plasma NFL elevation, which showed very high specificity (98% and 96%), can increase confidence in choosing drug-candidate patients. This may be an alternative way to identify patients in situations where PET scans are unavailable or patients have some contraindications to getting a lumbar puncture, such as coagulopathy.

We next investigated the ability of baseline plasma biomarker levels to predict future progression of drug candidate participants. Predicting future progression is important for clinical decision-making to identify older adults who would most benefit from therapeutic intervention. Identifying those likely to progress is also important for facilitating recruitment into prodromal AD clinical trials of new therapeutics. However, we found that plasma Aβ42/Aβ40 ratio, plasma p-tau181, and plasma NFL had poor performances (AUC 0.5–0.62) to predict clinical progression (Tables 3 and 4). On the other hand, hippocampal volume from structural imaging showed better performance over plasma biomarkers to predict clinical progression (AUC 0.62–0.71; Tables 3 and 4). Additionally, combined plasma p-tau181 and hippocampal atrophy at baseline showed high sensitivity but low specificity to predict cognitive worsening by CDRSB score progression.

These results were different from the results of the previous study from the BioFINDER cohort study (32), which showed that Plasma p-tau181 provided the best AUC (AUC 0.84) predicted CU to AD progression with AUC=0.84, while the combination of plasma p-tau181, pTau217, and Aβ42/Aβ40 had a high AUC of 0.87 to predict MCI to AD progression. The discrepancy in findings may be due to methodological differences. Our study of progression included only participants who were amyloid-positive and had cognitive impairment at baseline. In contrast, the previous study looked at a group of participants who had MCI or normal cognition, regardless of whether or not they had amyloid pathology. Secondly, the participants in the current study were only observed for 2 years after the baseline, while participants in the previous study were observed for 6 years. Therefore, it’s possible that the results of the current study could have been different if the observation period was longer.

In addition, we found that plasma Aβ42/Aβ40 had an inconsistent association with clinical progression in the cognitively impaired participants with amyloid pathology. Higher plasma Aβ42/Aβ40 levels were associated with faster worsening of CDRSB score over 2 years (Table 5), which we did not expect because there was strong evidence supporting that low plasma Aβ42 and Aβ42/Aβ40 ratio significantly correlated with brain amyloid burden (10, 26, 29). However, the result of our study for cognitive progression defined by diagnostic conversion was insignificant (Appendix 2). Although our cognitive change/clinical progression outcome measures (diagnosis and CDR-SB) were chosen for their clinical meaningfulness, future studies should evaluate the ability of plasma biomarkers and MRI to predict future decline in additional cognitive measures such as neuropsychological tests, some of which may be more responsive to change over shorter time periods.

In terms of the application of our results in different clinical settings, some caveats are warranted. Our cross-sectional results support the use of plasma biomarkers and structural MRI, which are less invasive and more easily accessible than PET scans, to help identify older adults for further screening who may be suitable candidates to receive anti-amyloid therapeutics. Since we included both CU and impaired participants in our analyses, these results can be applied to settings where doctors encounter patients with and without cognitive impairment, as geriatric clinics and primary care. However, these results may not generalize to memory clinics, where one would be unlikely to encounter participants without cognitive impairment. On the other hand, our longitudinal results, which evaluated the ability of plasma biomarkers and hippocampal atrophy at baseline to predict which drug-candidate participants are at risk for progressive cognitive decline, could be applied in a memory clinic for prioritizing and identify which patients are most likely to receive benefit from therapeutic intervention.

However, this study had limitations. First, there are a number of selection biases and a lack of diversity in ethnicity in the ADNI sample, which limits the generalizability of the findings. Second, the drug candidate participants in this study were mainly MCI participants; only 10.7% of them were mild AD. Thus, further studies for the drug candidate participants should enroll more AD participants. Third, renal function should be measured in further studies because it could alter plasma biomarker levels (33).

In conclusion, plasma Aβ42/Aβ40 was an alternative test that provided acceptable sensitivity for screening MCI and dementia with positive amyloid pathology patients. Combining plasma p-tau181 and structural brain imaging or plasma NFL with plasma Aβ42/Aβ40 provided very high specificity but low sensitivity in detection. In this study, hippocampal volume atrophy showed the best accuracy in predicting diagnosis conversion from MCI to dementia in the AD drug candidate participants.

## Supplementary material

Supplementary material, approximately 17.6 KB.

## Notes

*Ethical standards:* The ADNI protocols have been approved by all Institutional Review Boards of the participating institutions. The data used for these analyses were collected only from volunteers who provided written informed consent.
