# Supplementary material for: Performance of Plasma Biomarkers Combined with Structural MRI to Identify Candidate Participants for Alzheimer’s Disease-Modifying Therapy
Source: J Prev Alzheimers Dis. 2024 Jun 21;11(5):1198–205. doi: 10.14283/jpad.2024.110 (PMC11436390; doi:10.14283/jpad.2024.110)
Supplement: Supplementary file 1 — Supplementary material, approximately 17.6 KB. [file 42414_2024_110_MOESM1_ESM.docx]

**Supplementary material**

**Appendix 1**

Baseline characteristic comparison between non-progressors and progressors by CDRSB criteria of the drug candidate participants

| **Characteristic** | **Non-progressors**  **(n = 59)** | **Progressors**  **(n = 64)** | **P-value** |
| --- | --- | --- | --- |
| Age; median (IQR) | 74 (69, 79) | 73 (68, 77) | 0.3 |
| Diagnosis at baseline; n (%)   - MCI - Dementia | 51 (86%)  8 (14%) | 59 (92%)  5 (7.8%) | 0.3 |
| Male; n (%) | 1. 58%) | 37(58%) | >0.9 |
| Race; n (%)   - White - Nonwhite | 53 (90%)  6 (10%) | 62 (97%)  2 (3.1%) | 0.2 |
| Education years; median (IQR) | 16 (14, 18) | 16 (14, 18) | 0.5 |
| Married; n (%) | 42 (71%) | 53 (83%) | 0.2 |
| APOE4 status   - 0 allele - 1 allele - 2 allele | 26 (44%)  26 (44%)  7 (12%) | 18 (28%)  31 (48%)  15 (23%) | 0.1 |
| CDRSB scores; median (IQR) | 1 (0.5, 2) | 1.75 (1, 2.5) | 0.006 |
| Survival time; median (IQR) | 734 (727, 742) | 388 (214, 734) | <0.001 |
| Plasma Aβ_42_/Aβ_40_ ratio; median (IQR) | 0.109  (0.104, 0.116) | 0.115  (0.109, 0.121) | 0.002 |
| Plasma Aβ_42_/Aβ_40_ ratio ≤ 0.11 | 32 (54%) | 18 (28%) | 0.003 |
| Plasma p-tau181; median (IQR) | 17 (14, 26) | 21 (15, 27) | 0.14 |
| Plasma NFL; median (IQR) | 39 (27, 45) | 39 (29, 49) | 0.3 |
| Hippocampal volume at baseline;  Median (IQR) | 6868  (6288, 7815) | 6605  (5831, 7355) | 0.02 |

**Appendix 2**

Association between plasma biomarkers, hippocampal atrophy, and cognitive impairment progression by clinical diagnosis changing criteria; from MCI to dementia. (Total participant 109)

|  | **AF** | **95%CI** | **HR** | **95%CI** | **P-value** |
| --- | --- | --- | --- | --- | --- |
| Plasma Aβ_42_/Aβ_40_ (every 0.01 unit increasing) | 0.96 | 0.87-1.05 | 1.09 | 0.88-1.35 | 0.42 |
| Plasma NFL | 0.98 | 0.97-0.99 | 1.03 | 1.006-1.06 | 0.02 |
| Hippocampal volume (every 100 mm^3^ of shrinkage) | 1.05 | 1.02-1.07 | 0.91 | 0.87-0.94 | < 0.001 |
| Intracerebral volume | 0.99 | 0.99-1 | 1 | 0.99-1 | 0.27 |
| Age | 1.07 | 1.03-1.1 | 0.86 | 0.8-0.92 | < 0.001 |

AF: Acceleration Factor; HR: Hazard ratio
